# Neuroprotective effects of *Camellia nitidissima* Chi leaf extract in hydrogen peroxide‐treated human neuroblastoma cells and its molecule mechanisms

**DOI:** 10.1002/fsn3.1742

**Published:** 2020-07-22

**Authors:** Lei An, Wei Zhang, Guowei Ma, Ke Wang, Yufei Ji, Hong Ren, Yousheng Wang

**Affiliations:** ^1^ Beijing Advanced Innovation Center for Food Nutrition and Human Health Beijing Technology and Business University Beijing China

**Keywords:** antioxidant, *Camellia nitidissima* Chi, neurotrophic, SH‐SY5Y

## Abstract

*Camellia nitidissima* Chi (CNC) is a famous medicinal and edible plant with the name of “Tea for Longevity” in Guangxi province of China. In present study, we determined the protective effect of extract from CNC leaves on H_2_O_2_‐induced cell injury and its underlying mechanisms in human neuroblastoma (SH‐SY5Y) cells. The ethyl acetate fraction of CNC leaves (CLE, 50–200 μg/ml) treatment significantly increased the cell viability of H_2_O_2_‐treated SH‐SY5Y cells and reduced the leakage of LDH in a reversed “U”‐shape manner. It was confirmed by Hoechst 33,342 staining that CLE attenuated H_2_O_2_‐induced apoptosis in SH‐SY5Y cells. The CLE (100 and 150 μg/ml) treatment significantly relieved H_2_O_2_‐induced oxidative stress by decreasing intracellular ROS level, and increasing the activities of superoxide dismutase (SOD) and catalase (CAT). Western blot analysis demonstrated that the CLE treatment reserved H_2_O_2_‐induced decrease of pCREB (Ser133) expression, and its downstream protein BDNF. In addition, 37 phenolic compounds in CLE were identified by UPLC‐TOF MS/MS, and the main active phytochemicals seemed to be catechins, quercetin, kaempferol, and their derivatives. In conclusion, the data analysis showed that the neuroprotective effect of CNC leaves might be achieved via synergistically boosting endogenous antioxidant defenses and neurotrophic signaling pathway. These results suggest that CNC leaves are valuable resources for functional foods and beverages.

## Graphical abstract

In conclusion, the data analysis showed that the neuroprotective effect of *Camellia nitidissima* Chi leaf might be achieved via synergistically boosting endogenous antioxidant defenses and neurotrophic signaling pathway. These results suggest that CNC leaves are valuable resource for functional foods and beverages.

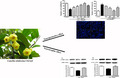

## 1 INTRODUCTION

*Camellia nitidissima* Chi (Theaceae family) is a famous ornamental and economic shrub with golden‐yellow flowers, which narrowly distributes in Guangxi Province, South China, and North Vietnam (Yang et al., 2008). It is a medicinal and edible plant and its flower, leaf, and seed oil show multiple health benefits, including antioxidant, anti‐inflammation, antitumor, antihyperlipidemic, and antidiabetic effects (Huang et al., 2009; Li et al., 2017; Yang, Guan, et al., 2018; Yang, Wang, Chen, & Jia, 2018). Recently, leaf and flower of CNC have attracted increasing attention for their bioactivities and potentials as functional food and drug (Hou et al., 2018; Wang et al., 2016).

As the elder population increasing, brain aging and its related neurodegenerative diseases, such as Alzheimer's disease has become a major burden of public health (Cutler, 2015). Among therapeutic and preventative remedies, one of the most prospective strategies is to boost brain's endogenous defenses by active phytochemicals. Excessive oxidative stress is considered to be a key player in age‐related brain pathology. The break of equilibrium between reactive oxygen species (ROS) and endogenous antioxidant defenses in brain has been reported to induce dystrophy and death of neurons, ultimately leading to the degeneration of brain function (Fagan‐Murphy, Hachoumi, Yeoman, & Patel, 2016; Halliwell, 1992).

In addition, loss of brain function in aging is attributed to weakening neuronal signaling that normally alleviate damage to brain cell (Poulose, Carey, & Shukitt‐Hale, 2007). cAMP‐response element binding protein (CREB) and its downstream target molecule brain‐derived neurotrophic factor (BDNF) are downregulated during oxidative stress damage in brain. It has been well documented that CREB and its downstream signaling pathway are impaired in brain aging conditions and neurodegenerative disorders (Gass & Riva, 2007; Saura & Valero, 2011; Zhu, Lau, Liu, Wei, & Lu, 2004). BDNF, one of the most important neurotrophic molecules, is considered as a key regulator involved in neuronal survival, differentiation, neurogenesis, as well as learning and memory (Lonze & Ginty, 2002). The decreased level of BDNF and pCREB was documented in both H_2_O_2_‐induced SH‐SY5Y cells in vitro and the brain of D‐galactose induced aged rats in vivo, while many phytochemicals such as polyphenols ameliorated these deficits (An et al., 2018; Qi et al., 2017; Shen, Xu, Qu, Sun, & Zhang, 2018; Yoo, Lee, Sok, Ma, & Kim, 2017). Increasing evidence suggests that CREB‐BDNF signaling pathway may be one of the common mechanisms boosting the brain's endogenous defenses by active phytochemicals.

In previous studies, we found that the ethanolic extract of CNC leaves demonstrated significant cytoprotection against H_2_O_2_‐induced SH‐SY5Y cells, which is one of the mostly validated cell models in neuroprotective effect evaluation (Feng et al., 2016; Park et al., 2015). However, the phytochemicals and mechanisms underlying the neuroprotective effect of CNC leaves are still unclear. Thus, in present study, we focus on further investigation of the neuroprotective effects of the CNC leaves, its mechanisms in endogenous defenses, as well as identifying the active compounds by UPLC‐TOF MS/MS.

## 2 MATERIALS AND METHODS

### 2.1 Materials

The leaves of *Camellia nitidissima* Chi (CNC) were collected in November 2016 from Fangchenggang, Guang xi, China, and authenticated by Mr. Liandong Huang. A voucher specimen (CNC‐2016‐11) of CNC was deposited in Dongxing Xinyu Industrial Co., Ltd. (Fangchenggang, China). All samples were frozen at −80°C for analysis.

Gallic acid and rutin (as standards) were purchased from Yuanye Bio Co., LTD (Shanghai, China). (±)‐6‐hydroxy‐2,5,7,8‐tetramethylchromane‐2‐carboxylic acid (Trolox) was obtained from Sigma‐Aldrich (China). 1,1‐diphenyl‐2‐picrylhydrazyl (DPPH) was purchased from TCI (Tokyo, Japan). 2,2′‐and‐bis (3‐ethylbenzothiazoline‐6‐sulfonic acid) diammonium salt (ABTS) and Folin–Ciocalteau reagent were purchased from Beijing Biotopped Co., LTD (Beijing, China). 2,4,6‐Tripyridyl‐S‐triazine (TPTZ) was obtained from Sinopharm Chemical Reagent Beijing Co., LTD, China. H_2_O_2_ was purchased from Wuhan Boster Biological Technology, Ltd. (Wuhan, China). Dulbecco's Modified Eagle's Medium (DMEM) media was obtained from Hyclone(Logan City, UT, USA), penicillin/streptomycin, trypsin‐EDTA were purchased from Gibco Life Technologies(Carlsbad, USA), fetal bovine serum (FBS) was obtained from Biological Industries(Israel), Lglutamine, sodium pyruvate and DCFH‐DA (2′, 7′‐dichlorodihydrofluorescin diacetate) were purchased from invitrogen (Carlsbad, USA). Hoechst 33,342 was purchased from Beyotime Biotechnology Institute (Nanjing, China). Lactate dehydrogenase (LDH) diagnostic kit, Superoxide Dismutase (SOD) activity, Catalase (CAT) assay kit, and BCA assay kit were obtained from Nanjing Jiancheng Bioengineering Institute (Nanjing, China). The anti‐pCREB (Ser133) and anti‐CREB were purchased from Merck Millipore (Germany). The anti‐BDNF were purchased from Sigma (USA). The secondary antibody HRP‐conjugated goat anti‐rabbit IgG was obtained from Applygen (Beijing, China). All solvents were of HPLC grade, and all chemicals were of analytical reagent grade.

### 2.2 Preparation of *Camellia nitidissima* Chi (CNC) leaf extract

The crushed leaves of CNC (200 g) were refluxed with 75% ethanol/water (4 L × 3) at 70°C for 1 hr, then combined and evaporated in a rotary evaporator at 45°C to yield the ethanolic extract (13.55 g). The suspended ethanolic extract (300 ml) was then extracted with n‐hexane（300 ml × 3）, ethyl acetate（300 ml × 3）, and n‐butanol (300 ml × 3), respectively. These fractions were then condensed and dried in vacuum freezing for 24 hr to obtain n‐hexane (3.03 g), ethyl acetate (4.49 g), and n‐butanol (3.43 g), and water (2.6 g) fractions. Phenolic contents and antioxidant activity measurement were described in Supporting Information.

### 2.3 Cell culture and treatment

Human neuroblastoma SH‑SY5Y cell line was kindly provided by Dr. You‐Zhi Zhang in Beijing Key laboratory of Neuropsychopharmacology, Institute of Pharmacology and Toxicology (Beijing, China) which originated from ATCC. The cells were cultured in DMEM media supplemented with heat inactivated 10% FBS, and 1% penicillin/streptomycin at 37°C in an atmosphere of 5% CO_2_.

To determine the appropriate damage concentration, cells (1 × 10^5^ cells/ml) were seeded in 96‐well plates. After 24 hr, the cells were incubated with different concentrations of H_2_O_2_ (100–800 μM) for 6 hr and then the cell viability was determined. To evaluate the neuroprotective effect of CNC samples, cells were divided into control group; H_2_O_2_ (200 μmol/L) group; H_2_O_2_ (200 μmol/L) plus CNC sample (50–200 μg/ml) groups. For the H_2_O_2_ plus CNC sample groups, cells were pretreated with CNC samples for 12 hr, and then treated with H_2_O_2_ for 6 hr. The extracts from CNC leaves were dissolved in DMSO, and the final concentration of DMSO was less than 0.1% (v/v).

### 2.4 Cell viability assay

Cell viability was evaluated by MTT assay. 10 μl of MTT solution (5 mg/ml) in PBS was added to each well. After incubation at 37°C for 4 hr, 90 μl DMSO was added to dissolve the formazan. 96‐well plates were agitated on a microtiter plate for 10 min, and the optical densities (OD) were read at 570 nm using a microplate reader (INFINITE M1000 P120, Tecan, Mannedorf, Switzerland). The results were expressed as a relative percentage of the control group according to equation: Relative Cell viability (%) = (OD of treatment group − OD of blank group)/(OD of control group − OD of blank group) × 100%.

### 2.5 Determination of Hoechst 33,342 staining

To observe cell apoptosis, cells (1 × 10^5^ cells/ml) were seeded in 6‐well plates. After 24 hr, the cells were treated with different concentrations of CLE for 12 hr. Subsequently, the cells were treated with H_2_O_2_ for 6 hr, and then the medium was replaced with Hoechst 33,342 solution and incubated at 37°C for 10 min. It was then washed 3 times with PBS to remove excess dye. Cells were observed under a fluorescence microscope (Axioplan 2 imaging E, Carl Zeiss, Germany). Apoptotic cells exhibit strong blue fluorescence and atrophic nuclei, while nonapoptotic cells exhibit weak blue fluorescence and normal nuclei.

### 2.6 Determination of extracellular Lactate dehydrogenase (LDH) activity

The cell damage was judged by the level of LDH in the cell culture medium. The SH‐SY5Y cells were cultured in 24‐well plates, and after the treatment, the supernatants were collected. The amount of LDH in the medium was determined by the LDH assay kit.

### 2.7 Determination of intracellular ROS

The intracellular ROS level was determined by DCFH‐DA that will be oxidized by intracellular ROS. SH‐SY5Y cells 1 × (10^5^ cells/ml) were seeded in black 96‐well plates. After the H_2_O_2_ (200 μM) treatment, the cells were washed with PBS and incubated with 100 μl of DCFH‐DA (20 μM) in dark at 37°C for 40 min. The cells were washed with PBS for 3 times, and fluorescence was measured by a fluorescence microplate reader (INFINITE M1000 P120, Tecan, Mannedorf, Switzerland) at an excitation wavelength of 500 nm and an emission wavelength of 530 nm. The ROS levels were expressed as a relative percentage of the control.

### 2.8 Determination of SOD and CAT activity

The activity of antioxidant enzymes, SOD and CAT, was measured according to the manufacturer's instructions. After the treatment, cells were washed with PBS and then lysed with cell lysis buffer in ice bath for 30 min. The homogenate was centrifuged at 3000 ×*g* for 15 min, and protein concentration of the supernatant was determined by bicinchoninic acid (BCA) method. The supernatant was then collected for SOD and CAT measurement. The SOD assay is based on the characteristic absorption (A) of nitrite at 550 nm, and the CAT assay was determined by detecting the characteristic absorption of complex compound at 405 nm. The activity of SOD and CAT was calculated by equations: SOD activity (U/mg prot) = (A of control tube − A of testing tube) × 2/A of control tube × reaction volume/(sample volume × ptotein concentration); CAT activity (U/mg prot) = (A of control tube − A of testing tube) × 271/(60 × sample volume × ptotein concentration).

### 2.9 Western blot analysis

Cells were collected, washed with PBS, lysed using whole cell lysates (RIPA buffer with protease inhibitor cocktail and phosphatase inhibitors), homogenized and centrifuged. The protein concentration was determined by BCA assay kit. For Western blot analysis, samples (70 μg protein) were separated in SDS‐polyacrylamide gels and transferred to nitrocellulose membranes. Membranes were blocked with 5% defatted milk at room temperature for 2 hr and then incubated with the following primary antibodies of rabbit anti‐BDNF (1:1,000) or anti‐pCREB (1:1,000) at 4°C overnight. The membranes were then washed with 0.1 M Tris‐buffered (10 min × 3) and incubated with secondary antibody HRP‐conjugated goat anti‐rabbit IgG (1:2,500) for 2 hr at room temperature. The protein band was developed by a Super Signal kit, and the chemiluminescence signal was transformed into a digital image. β‐Actin was used as a control marker.

### 2.10 UPLC‐TOF MS/MS

The ethyl acetate fraction of CNC leaves (CLE) was used for phenolic compounds identification by UPLC‐QTOF‐MS/MS method. The UPLC‐Q‐TOF‐MS/MS (Agilent Technologies 6,530 Accurate‐Mass Q‐TOF LC/MS) was equipped with an Agilent Proshell 120 EC‐18 (4.6 × 100 mm, 2.7 µm) column. The mobile phase included A: 95% methanol and B: 0.2% formic acid in water. The gradient of UPLC was followed: 0–10 min, 5%‐25% A; 10–20 min, 25%–55% A; 20–25 min, 55%–80% A; 25–30 min, 80%–100% A; 30–35 min, 100%–50% A; 35–40 min, 50%–25% A; 40–43 min, 25%–5% A; 43–50 min, 5% A. The sample injection volume was 10 µl and the flow rate was 0.5 ml/min. The temperature was set at 35°C. The Q‐TOF‐MS/MS system was used with the following parameters: capillary voltage 4 kV, fragmentor 120 V, ion source heater temperature 300°C, curtain gas 35 psi and collision energy 20 eV. The full scan mass spectra data were collected at 100–1,000 m/z and fragments mass spectra 50–1,000 m/z under the negative ion scanning condition mode. The mass and mass‐mass data were processed with the Masshunter software (Agilent Technologies).

### 2.11 Data analysis

Results were expressed as means ± standard deviation (*SD*) and analyzed by ANOVA followed by Dennett's *t* test for intergroup comparisons (GraphPad Prism 6.0, GraphPad software Inc., San Diego, CA). A value of *p* < .05 was considered to be significant.

## 3 RESULTS AND DISCUSSION

### 3.1 Ethyl acetate fraction from *Camellia nitidissima* Chi leaves (CLE) inhibited H_2_O_2_‐induced injury in SH‐SY5Y cells

**FIGURE 1:**
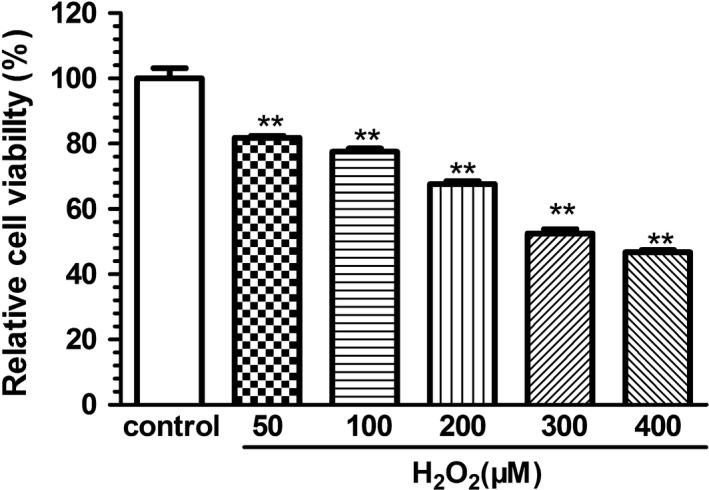
Effect of H_2_O_2_ on SH‐SY5Y cell viability by MTT assay. Each column is expressed as mean ± S.E.*M* (*n* = 6). Data are expressed as a relative percentage of the control. One‐way ANOVA followed by Dunnett's test was used for statistical analysis, **p < .01, compared with control group. Results are representative of at least three individual experiments

As shown in Figure 1, incubation with H_2_O_2_ (50~400 μM) for 6 hr significantly decreased the cell viability in a concentration‐dependent manner. And H_2_O_2_ at the concentration of 200 μmol/L decreased the cell viability to approximately 60%, which was considered suitable for cytoprotection and mechanism research (Nopparat, Chantadul, Permpoonputtana, & Govitrapong, 2017; Yang et al., 2014), and thus, this concentration was used in subsequent experiments.

**FIGURE 2:**
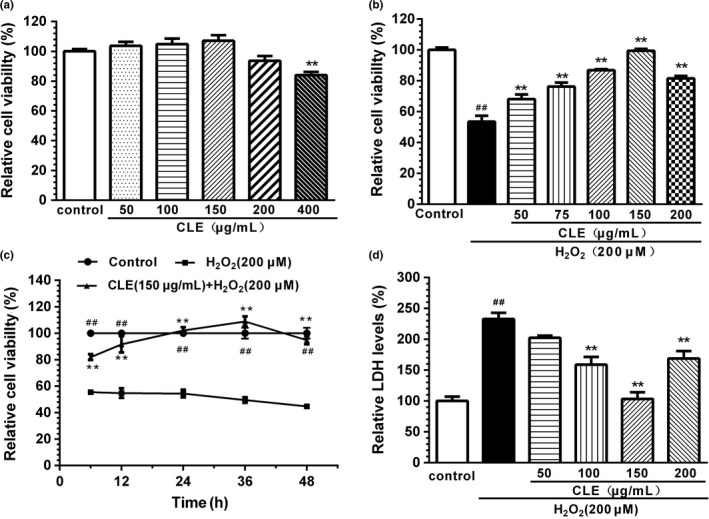
The effect of CLE on H_2_O_2_‐induced injury in SH‐SY5Y cells. (a) Effect of CLE on SH‐SY5Y cell viability by MTT assay. (b) Effect of CLE on viability of H_2_O_2_‐treated SH‐SY5Y cells by MTT assay. (c) Effect of CLE treatment on the viability of H_2_O_2_‐treated SH‐SY5Y cells at 6, 12, 24, 36, or 48 hr. (d) Cell viabilities according to LDH leakage assays. Each column is expressed as mean ± S.E.*M* (*n* = 6). Data are expressed as a relative percentage of the control. CLE: ethyl acetate fraction from *C. nitidissima* Chi leaves. The effect of CLE treatment at different time point was analyzed by repeated measures ANOVA, while other tests were analyzed by One‐way ANOVA (Dunnett's test), ^##^ *p* < .01, compared with respective control group, *p < .05, **p < .01, compared with respective H_2_O_2_ group. Results are representative of at least three individual experiment

Firstly, we evaluated the phenolic content, antioxidant activity (DPPH, ABTS, and FRAP) in vitro and cytoprotection of CNC leaf extract and its four fractions. Ethyl acetate fraction (CLE) demonstrated the best protective effect against H_2_O_2‐_induced cell death (see results in Figure S1), as well as the highest total phenolic content (Table S1) and antioxidant activity (Table S2), in comparison with other fractions. We then evaluated the effect of CLE on the cell viability of non‐H_2_O_2_‐treated and H_2_O_2_‐treated SH‐SY5Y cells. Cells were incubated with CLE (50~400 μg/ml) for 12 hr, and then, the cell viability was determined by MTT assay. The result was shown in Figure 2a, and CLE treatment had no significant effect on cell viability at the concentrations from 50 to 200 μg/ml, although a slight decrease with 200 μg/ml. Higher concentration of CLE (400 μg/ml) significantly decreased the cell viability to approximately 84% (*p* < .01), compared with control, indicating this concentration is harmful for the cell survival. Then, we chose the concentration of CLE from 50 to 200 μg/ml for further determination. As shown in Figure 2b, pretreatment with CLE (50~200 μg/ml) for 12 hr significantly increased the cell viability (*p* < .01) in a reversed “U”‐shape manner. CLE at the concentration of 150 μg/ml showed the strongest effect, increased the cell viability from 53.5% (H_2_O_2_‐treated group) to 99.4% (*p* < .01). We also determined the effect of CLE treatment on the viability of H_2_O_2_‐treated SH‐SY5Y cells at 6, 12, 24, 36, or 48 hr. The results (Figure 2c) showed that CLE exerted significantly cytoprotective effect from 6 to 48 hr. The protection of CLE was also confirmed by detecting LDH leakage (Figure 2d). CLE (100~200 mg/ml) treatment significantly reduced H_2_O_2_‐induced LDH leakage (*p* < .01). Similar to the cell viability results, CLE at the concentration of 150 μg/ml showed the strongest effect, decreased the extracellular LDH level from 232.94% to 103.32% (*p* < .01).

**FIGURE 3:**
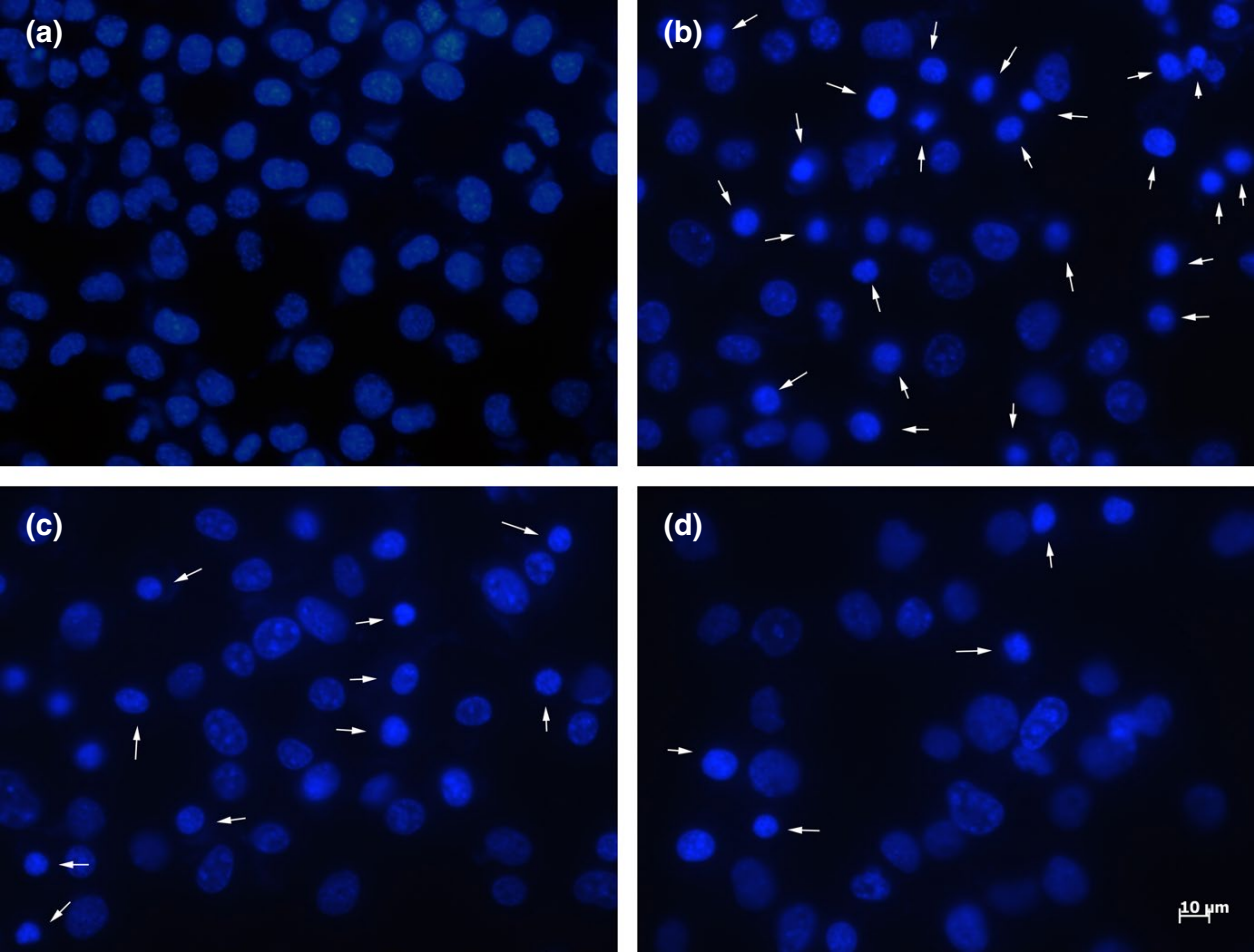
Representative Hoechst 33,342 staining images of cell apoptosis. (a) control, (b) 200 μM H_2_O_2_, (c) 100 μg/ml CLE + 200 μM H_2_O_2_, (d) 150 μg/ml CLE + 200 μM H_2_O_2_. White arrows indicated apoptotic nuclei

To further investigate the cytoprotective effect of CLE, the apoptotic changes of SH‐SY5Y cells were detected by Hoechst 33,342 staining. The results showed that H_2_O_2_ treatment increased the number of apoptotic cells, demonstrating nuclear chromatin condensation and fragmentation in Figure 3, and the CLE treatment attenuated H_2_O_2_‐induced changes in SH‐SY5Y cell, normalizing nuclear chromatin morphology. These results indicate that CLE demonstrated significant cytoprotective effect against H_2_O_2_‐treatment. The data also showed us a U‐shaped dose‐effect curve that coincided with the cell viability results.

### 3.2 CLE relieved H_2_O_2_‐induced oxidative stress in SH‐SY5Y cells

**FIGURE 4:**
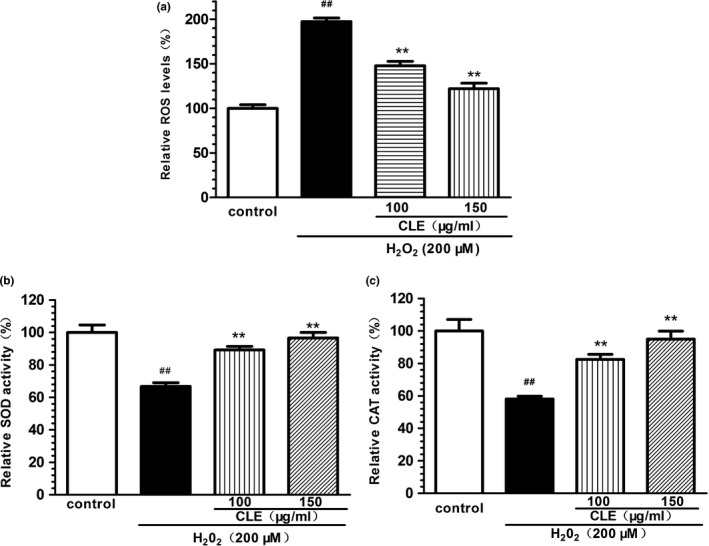
Effect of CLE on H_2_O_2_‐induced oxidative stress in SH‐SY5Y cells (a) Effect of CLE on the levels of ROS in H_2_O_2_‐treated SH‐SY5Y cells. (b) Effect of CLE on the activity of SOD in H_2_O_2_‐treated SH‐SY5Y cells. (c) Effect of CLE on the activity of CAT in H_2_O_2_‐treated SH‐SY5Y cells. CLE: ethyl acetate fraction from *C. nitidissima* Chi leaves. Each column is expressed as mean ± S.E.*M* (*n* = 6). Data are expressed as a relative percentage of the control. One‐way ANOVA followed by Dunnett's test was used for statistical analysis, ^##^ *p* < .01, compared with control group, **p < .01, compared with H_2_O_2_ group. Results are representative of at least three individual experiments

Excess addition of H_2_O_2_ can induce intracellular ROS production, an important indicator of oxidative stress, which may affect endogenous antioxidant defenses, metabolic responses and signaling pathways within cells, and even cause cell death. As shown in Figure 4a, treatment with 200 μM H_2_O_2_ increased ROS level by almost twofold in SH‐SY5Y cells (*p* < .01). Pretreatment with CLE (100 and 150 μg/ml) significantly reduced the ROS level (*p* < .01), which may be one of the mechanisms underlying the cytoprotective effect of CLE.

As important members of endogenous antioxidant defenses, SOD and CAT prevent the cell from oxidative stress. In present study, incubation with H_2_O_2_ significantly reduced the activity of SOD and CAT to 67.9% and 59.4% of the control group, respectively (Figure 4b,c). CLE pretreatment with 100 and 150 μg/ml attenuated the decreased activity of SOD and CAT (*p* < .01). These results indicated that CLE treatment increased the endogenous antioxidant defenses in H_2_O_2_‐treated SH‐SY5Y cells.

### 3.3 CLE increased pCREB and BDNF expression in H_2_O_2_‐treated SH‐SY5Y cells

**FIGURE 5:**
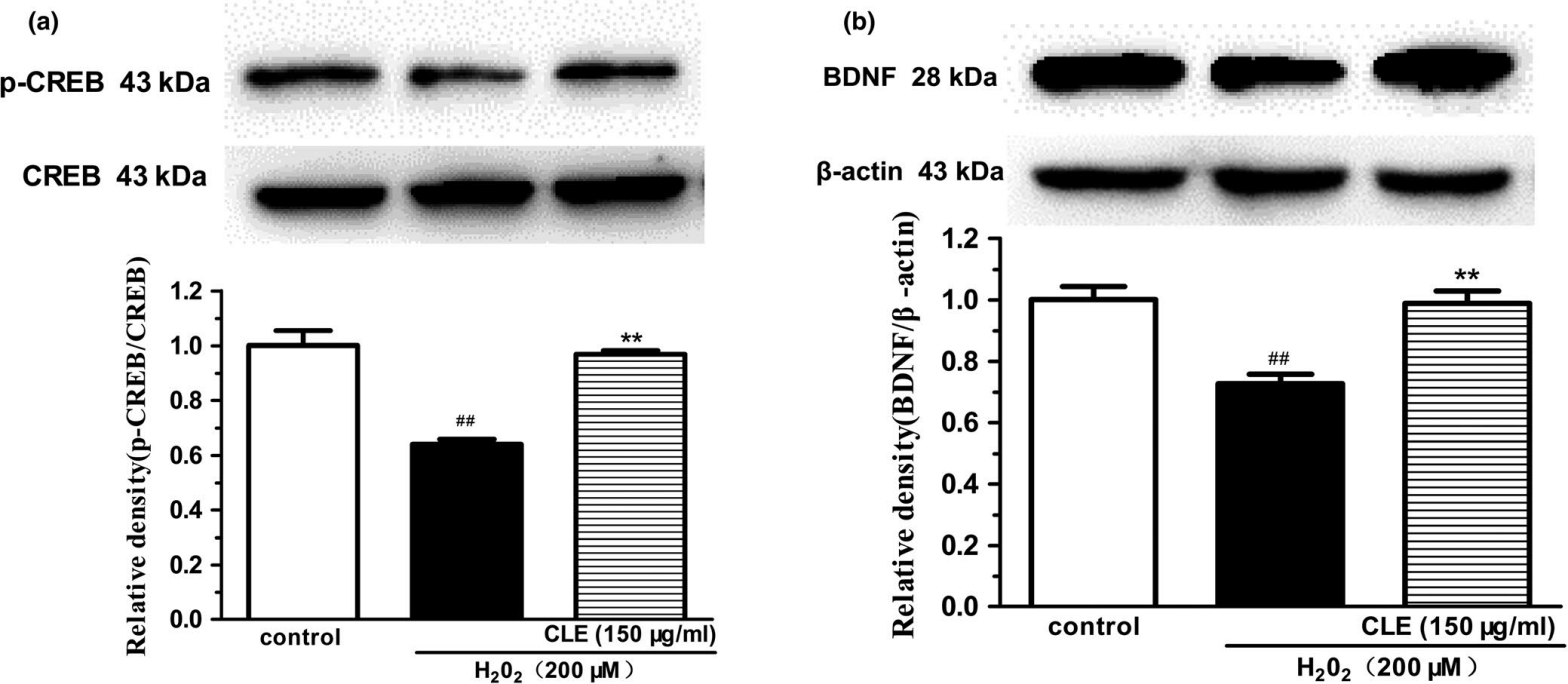
Effect of CLE on pCREB and BDNF expression in H_2_O_2_‐treated SH‐SY5Y cells. The intensity of western bands was quantified with a densitometric scanner. CLE: ethyl acetate fraction from *C. nitidissima* Chi leaves. Each column is expressed as mean ± *SEM* (*n* = 3). ^##^ *p* < .01, compared with control group. **p < .01, compared with H_2_O_2_ group (ANOVA followed by Dunnett's test). Results are representative of at least three individual experiments

Western blot (Figure 5a) showed that H_2_O_2_ treatment significantly decreased pCREB expression to approximately 64.0% of the control (*p* < .01). CLE (150 μg/ml) treatment reversed this change and significantly increased pCREB expression (*p* < .01). A similar result was obtained for BDNF expression (Figure 5b). These results suggest that CLE treatment could produce potent neurotrophic effects and upregulate the CREB‐BDNF signaling in SH‐SY5Y cells.

### 3.4 Active compounds analysis in CLE by UPLC‐TOF MS/MS

**FIGURE 6:**
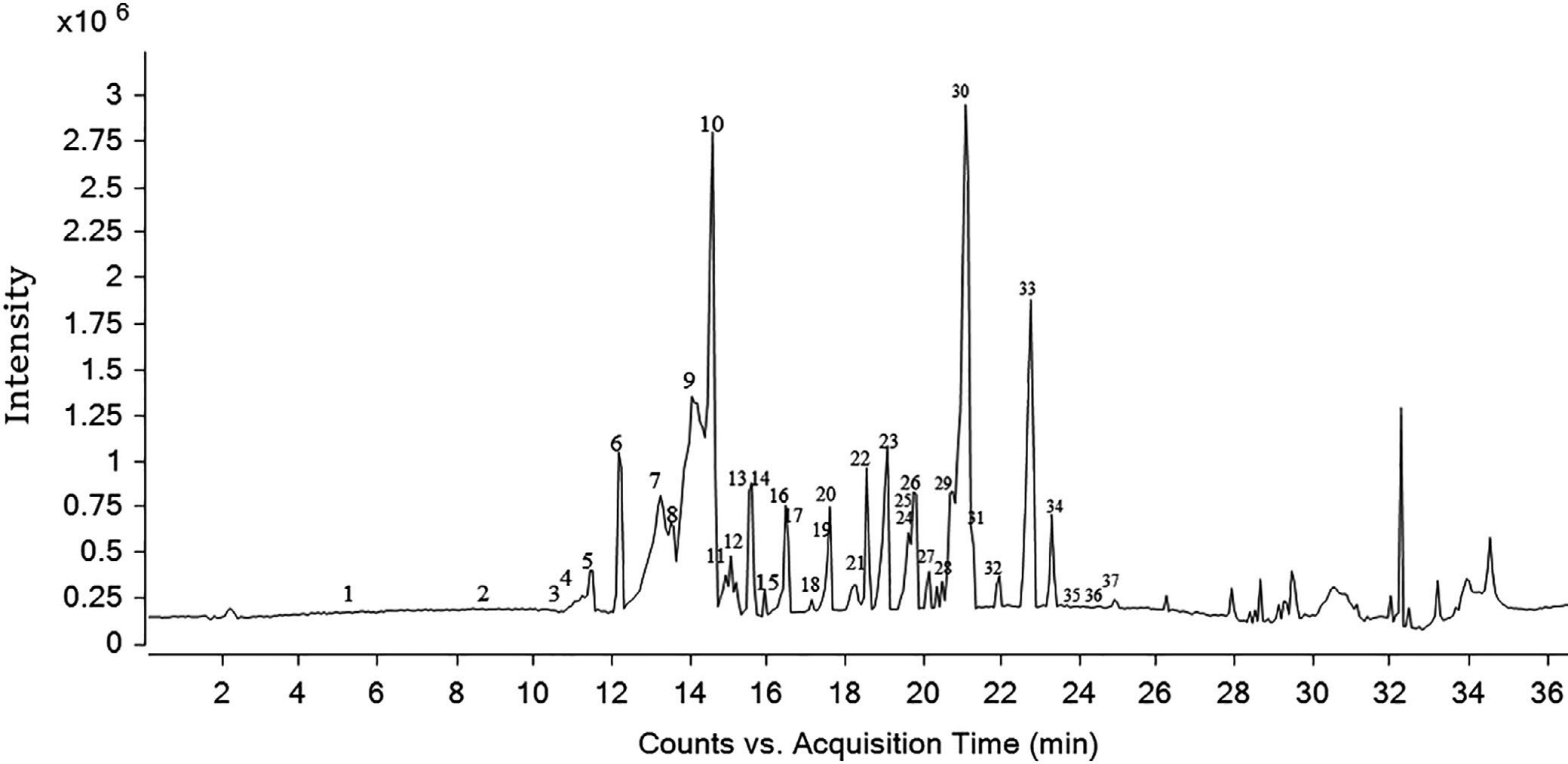
UPLC‐Q‐TOF total ion chromatogram of ethyl acetate fraction of *C. nitidissima* Chi leaves

Thirty‐seven phenolic compounds were identified in CLE (Table 1) by UPLC‐TOF MS/MS method (Figure 6).

**TABLE 1:** Phenolic compounds in ethyl acetate fraction from *C. nitidissima* Chi leaves by UPLC‐Q‐TOF‐Mass HunterPeak

	Compound	Molecular formula	Measured (M‐H)^−^ (m/z)	MS/MS	Rt (min)	Reference	Relative content^a^ (%)
1	Gallic acid	C_7_H_6_O_5_	169.0136	125	5.354	Yang, Guan, et al. (2018), Xie et al. (2017), Baskaran et al. (2016)	0.88
2	Protocatechuic acid	C_7_H_6_O_4_	153.0191	109	8.5	Baskaran et al., (2016)	2.82
3	Catechin dimmer or procyanidin dimer	C_30_H_26_O_12_	577.1341	289 407 425 451	11.245	Wang et al. (2016), Yang, Guan, et al. (2018), Baskaran et al. (2016)	42.83
4	Catechin	C_15_H_14_O_6_	289.0713	109 123 137 151 203 245	11.379	Wang et al. (2016), Yang, Guan, et al. (2018), Baskaran et al., (2016)	4.66
5	*p*‐Coumaroyl‐quinic acid	C_16_H_18_O_8_	337.0962	119 163	11.513		25.87
6	catechin dimmer or procyanidin dimer	C_30_H_26_O_12_	577.1341	289 407 425 451	12.182	Wang et al. (2016), Yang, Guan, et al. (2018), Baskaran et al. (2016)	26.52
7	(E)Afz–(E)C(1)	C_30_H_26_O_11_	561.1452	289 407 435	13.253	Song et al. (2011)	100.00
8	Procyanidin trimer	C_45_H_38_O_18_	865.1975	125 287 289 425 577 695 713	13.722	Baskaran et al. (2016)	3.24
9	(E)Afz–(E)C(2)	C_30_H_26_O_11_	561.1452	289 407 435	14.057	Song et al. (2011)	34.28
10	Epicatechin	C_15_H_14_O_6_	289.0713	109 123 137 151 203 245	14.592	Wang et al. (2016), Yang, Guan, et al. (2018), Baskaran et al. (2016)	70.17
11	(E)Afz–(E)C–(E)C	C_45_H_38_O_17_	849.2019	289 407 559	15.061	Song et al. (2011)	5.18
12	(E)Afz–(E)Afz–(E)C (1)	C_45_H_38_O_16_	833.2161	289 543 561 707	14.927	Song et al. (2011)	32.34
13	Dihydroquercetin galactoside	C_21_H_22_O_12_	465.1082	125 151 285 303	15.596	Jia et al. (2016)	11.46
14	(E)Afz–(E)Afz–(E)C (2)	C_45_H_38_O_16_	833.2161	289 543 561 707	15.596	Song et al. (2011)	23.17
15	(E)Afz–(E)Afz–(E)Afz–(E)C	C_60_H_50_O_21_	1,105.2865	271 289 543 815 833	15.931	Song et al. (2011)	3.74
16	Afzelechin	C_15_H_14_O_5_	273.0768	107 135 137	16.467	Jia et al. (2016)	27.00
17	Catechin dimmer or procyanidin dimer	C_30_H_26_O_12_	577.1341	289 407 425 451	16.801	Wang et al. (2016); Yang, Guan, et al. (2018), Baskaran et al. (2016)	5.65
18	Catechin dimmer or procyanidin dimer	C_30_H_26_O_12_	577.1341	289 407 425 451	17.136	Wang et al. (2016), Yang, Guan, et al. (2018), Baskaran et al. (2016)	5.07
19	Dihydrokaempferol‐galactoside	C_21_H_22_O_11_	449.1084	125 151 269 287	17.605		24.84
20	Dihydroquercetin glucoside	C_21_H_22_O_12_	465.1082	125 151 285 303	17.873	Wang et al. (2016)	3.51
21	(E)Afz–(E)C(3)	C_30_H_26_O_11_	561.1452	289 407 435	18.274	Song et al. (2011)	16.95
22	(E)Afz–(E)C(4)	C_30_H_26_O_11_	561.1452	289 407 435	18.542	Song et al. (2011)	16.95
23	Dihydroquercetin‐rhamnoside	C_21_H_22_O_11_	449.1089	125 151 285 303	19.078	Wang et al. (2016), Song et al. (2011)	37.73
24	Quercetin‐glucoside‐rhamnoside	C_27_H_30_O_16_	609.1462	151 179 300 301 463	19.679	Xie et al. (2017)	4.26
25	(E)Afz–(E)Afz–(E)C (3)	C_45_H_38_O_16_	833.2161	289 543 561 707	19.68	Song et al. (2011)	2.07
26	Quercetin‐galactoside	C_21_H_20_O_12_	463.0881	151 301	19.747	Wang et al. (2016)	38.42
27	Quercetin pentoside	C_20_H_18_O_11_	433.0818	151 301	20.149	Song et al. (2011)	17.99
28	Dihydrokaempferol‐rhamnoside	C_21_H_22_O_10_	433.117	125 151 269 287	20.751		23.19
29	Quercetin‐rhamnoside (Quercitrin)	C_21_H_20_O_11_	447.0928	151 301	21.019	Song et al. (2011)	83.04
30	Kaempferol‐rutinoside	C_27_H_30_O_15_	593.1511	285	21.287	Wang et al. (2016), Yang, Guan, et al. (2018)	2.50
31	Isoscutellarin	C_22_H_22_O_11_	461.1128	163 297	21.421	Xie et al. (2017)	2.21
32	Quercetin‐glucoside	C_21_H_20_O_12_	463.0881	151 301	22.022	Wang et al. (2016)	0.6
33	Kaempferol‐rhamnoside	C_21_H_20_O_10_	431.0983	285,151	22.759		53.04
34	Quercetin	C_15_H_10_O_7_	301.0352	151 179 273	23.295	Yang, Guan, et al. (2018)	16.07
35	Quercetin‐3‐O‐[2‐O‐(6‐O‐p‐hydroxyl‐E‐coumaroyl)‐D‐glucosyl]‐(1‐2)‐L‐rhamnoside	C_36_H_36_O_18_	755.1826	151 301 476 609	23.764		0.83
36	Epiafzelechin	C_15_H_14_O_5_	273.0768	107 137	24.366	Jia et al. (2016)	0.60
37	Kaempferol	C_15_H_10_O_6_	285.0405	159 187 211 239	24.902	Yang, Guan, et al. (2018), Jia et al. (2016)	5.09

^a^ The area of seventh peak [(E) Afz–(E)C(1)] is considered as 100%.

Peak 1 (Rt 5.354 min) with the extract ion chromatogram at m/z 169.0136 showed the fragment at m/z 125, which was corresponding to the loss of COO^−^. It was identified as gallic acid. Peak 2 (Rt 8.5 min) with the extract ion chromatogram at m/z 153.0191 showed the fragment at m/z 109, which was corresponding to the loss of COO^−^. It was identified as protocatechuic acid (Baskaran, Pullencheri, & Somasundaram, 2016). Peak 5 (Rt 11.513 min) with the extract ion chromatogram at m/z 337.0962 demonstrated the fragments at m/z 119 and 163, which was corresponding to the loss of C_8_H_11_O_7_ and C_7_H_11_O_5_, respectively. The peak was considered as p‐Coumaroyl‐quinic acid (Baskaran et al., 2016; Xie et al., 2017; Yang, Guan, et al., 2018).

Peak 4 and 10 (Rt 11.379 and 14.592 min) with the extract ion chromatogram at m/z 289.0713 demonstrated the fragments at m/z 109, 123, 137, 151, 203, and 245, which was corresponding to the loss of C_10_H_12_O_3_, C_9_H_10_O_3_, C_8_H_8_O_3_, C_7_H_6_O_3_, C_4_H_6_O_2,_ and CO_2_, respectively. They were identified as catechin (Rt 11.379) and epicatechin (Rt 14.592) (Baskaran et al., 2016; Wang et al., 2016; Yang, Guan, et al., 2018). Peak 3, 6, 17, and 18 (Rt 11.245, 12.182, 16.801, and 17.136 min) with the extract ion chromatogram at m/z 577.1341 demonstrated the fragments at m/z 289, 407, 425, and 451, which was corresponding to the loss of C_15_H_12_O_6_, C_8_H_10_O_4_, C_8_H_8_O_3,_ and C_6_H_6_O_3_, respectively. These peaks were considered to be catechin dimmer or procyanidin dimer (Wang et al., 2016; Yang et al., 2008; Yang, Guan, et al., 2018). Peak 8 (Rt 13.722 min) with the extract ion chromatogram at m/z 865.1975 demonstrated the fragments at m/z 125, 287, 289, 425, 577, 695, and 713, which was judged to be [M‐H‐288‐288]^−^. It was identified as procyanidin trimer (Baskaran et al., 2016).

Peak 13, 20, and 23 (Rt 15.596, 17.873, and 19.078 min) with the extract ion chromatogram at m/z 465.1082 and 449.1089 demonstrated the fragments at m/z 125, 151, 285, and 303, which were considered to be [M‐H‐2‐162]^−^, [M‐H‐2‐162]^−^ and [M‐H‐2‐146]^−^, respectively. They were identified as dihydroquercetin glucoside (Rt 15.596 min), dihydroquercetin glucoside (Rt 17.873 min), and dihydroquercetin‐rhamnoside (Rt 19.078 min) (Song, Wang, Zheng, & Huang, 2011; Wang et al., 2016).

Peak 19 and 28 (Rt 17.605 and 20.751 min) with the extract ion chromatogram at m/z 449.1084 and 433.117 demonstrated the fragments at m/z 125, 151, 269, 287. The fragment at 125 m/z was characteristic of dihydroflavonols. It was reported that the fragments of dihydrokaempferol were at m/z 269, 259, 151, and 125 (Wang et al., 2016), and the fragment at m/z 285 was considered to be [M‐H‐2‐162]^−^ and [M‐H‐2‐146]^−^. So peak 19 and 28 were identified as dihydrokaempferol‐galactoside and dihydrokaempferol‐rhamnoside, respectively.

Peak 31 and 36 (Rt 21.421 and 24.366 min) with the extract ion chromatogram at m/z 273.0768 demonstrated the fragments at m/z 107, 135, and 137, which was corresponding to the loss of C_9_H_10_O_3_, C_8_H_10_O_2_, and C_8_H_8_O_2_, respectively. These peaks were considered as afzelechin (Rt 21.421 min) and epiafzelechin (Rt 24.366 min) (Jia et al., 2016). Peak 7, 9, 21 and 22 (Rt 13.253, 14.057, 18.274, and 18.542 min) with the extract ion chromatogram at m/z 561.1452 demonstrated the fragments at m/z 289, 407, and 435, which were considered to be [M‐H‐289]^−^. These peaks were identified as (E)Afz–(E)C (Song et al., 2011). Peak 12, 14, and 25 (Rt 14.927, 15.596, and 19.68 min) with the extract ion chromatogram at m/z 833.2161 demonstrated the fragments at m/z 289, 543, 561, and 707, which were considered to be [M‐H‐273–289]^−^. These peaks were identified as (E)Afz–(E)Afz–(E)C. Peak 11 (Rt 15.061 min) with the extract ion chromatogram at m/z 849.2019 showed the fragment at m/z 289, 407, and 559, which were considered to be [M‐H‐289–289]^−^. It was identified as (E)Afz–(E)C–(E)C (Song et al., 2011). Peak 15 (Rt 15.931 min) with the extract ion chromatogram at m/z 1,105.2865 showed the fragments at m/z 271, 289, 543, 815, and 833, which were considered to be [M‐H‐273–273–289]^−^. It was identified as (E)Afz–(E)Afz–(E)Afz–(E)C (Song et al., 2011).

Peak 34 (Rt 23.295 min) with the extract ion chromatogram at m/z 301.0352 demonstrated the fragments at m/z 151, 179 and 273, which was corresponding to the loss of C_8_H_6_O_3_, C_7_H_6_O_2_, and one H_2_O, respectively. It was identified as quercetin (Yang, Guan, et al., 2018). Peak 29 (Rt 21.019 min) with the extract ion chromatogram at m/z 447.0928 demonstrated the fragments at m/z 301 [M‐H‐146]^−^ and 151. This compound was identified as quercetin‐rhamnoside. Peak 26 and 32 (Rt 19.747 and 22.022 min) with the extract ion chromatogram at m/z 463.0881 demonstrates the fragments at m/z 301 [M‐H‐162]^−^ and 151. The two peaks were identified as quercetin‐galactoside (Rt 19.747 min) and quercetin‐glucoside (Rt 22.022 min) (Wang et al., 2016). Peak 27, 24, and 35 (Rt 20.149, 19.679 and 23.764 min) with the extract ion chromatogram at m/z 433.0776, 609.1462, and 755.1826, all demonstrated the fragment at m/z 285 which was considered to be [M‐H‐132]^−^, [M‐H‐162‐146]^−^ and [M‐H‐146‐162‐146]^−^, respectively. They were identified asquercetin pentoside, quercetin‐glucoside‐rhamnoside (Xie et al., 2017), and quercetin‐3‐O‐[2‐O‐(6‐O‐p‐hydroxyl‐E‐coumaroyl)‐D‐glucosyl]‐(1‐2)‐L‐rhamnoside, respectively.

Peak 37 (Rt 24.902 min) with the extract ion chromatogram at m/z 285.0391 demonstrated the fragments at m/z 159, 187, 211, and 239. This peak was identified as kaempferol (Jia et al., 2016; Yang, Guan, et al., 2018). Peak 33 and 30 (Rt 22.759 and 21.287 min) with the extract ion chromatogram at m/z 431.0983 and 593.1511 demonstrated the fragment at m/z 285 which was considered to be [M‐H‐146]^−^ and [M‐H‐146‐162]^−^, respectively. They were identified as kaempferol‐rhamnoside and kaempferol‐rutinoside, respectively (Wang et al., 2016; Yang, Guan, et al., 2018).

The relative contents of active compounds in CLE were determined by area nomalization method. The result (showed in Table 1) demonstrated the main phytochemicals were catechin, quercetin, kaempferol, and its derivatives.

## 4 CONCLUSION

It is well‐known that natural products are important sources of neuroprotective agents (Babaei et al., 2020; Koushki, Amiri‐Dashatan, Ahmadi, Abbaszadeh, & Rezaei‐Tavirani, 2018). In the present study, we determined the neuroprotective effect and the underlying mechanisms of extract in CNC leaves. Data in present study demonstrated that (1) the treatment with the ethyl acetate fraction of CNC leaf extract (CLE, 50–200 μg/ml) significantly inhibited H_2_O_2_ **‐**induced death of SH‐SY5Y cells; (2) the CLE treatment significantly reversed H_2_O_2_ **‐**induced oxidative stress and decrease of CREB‐BDNF neurotrophic signaling; (3) the main active phytochemicals might be catechin, quercetin, procyanidin, kaempferol and their derivatives.

The overproduction of free radicals and oxidative stress has been linked to brain aging and several neurodegenerative diseases including cognitive dysfunction and Alzheimer's disease. In present study, we determined and compared the phenolic contents and antioxidant activity of the different fractions of CNC leaves, as well as their cytoprotection against H_2_O_2_‐treated SH‐SY5Y cells. CLE demonstrated the best protective effect against H_2_O_2_ **‐**induced cell death, as well as the highest total phenolic content and antioxidant activity in vitro.

Accumulated evidence revealed that overproduction of ROS disrupted not only the balance between oxidation and antioxidant defenses, but also protective neuronal signaling, and ultimately to disrupt structures and function of brain cells. Among the overproduction of free radicals, H_2_O_2_ is considered to be one of the most important ROS species that cause these damage (Yang et al., 2016). In present study, we used H_2_O_2_‐treated SH‐SY5Y cells to mimic this process and evaluate the neuroprotective effect of CNC leaves. Our data demonstrated that the treatment with CLE at concentration of 50 to 200 μg/ml significantly relieved H_2_O_2_ **‐**induced cell injury and oxidative stress. Moreover, we found that H_2_O_2_‐treatment significantly decreased the CREB‐BDNF signaling pathway in SH‐SY5Y cells, which is normally supposed to exert neurotrophic effect and alleviate damage to brain cells. While CLE treatment reversed this downregulation. These results suggested that the neuroprotective effect might be attributed to both the neurotrophic effect and the antioxidative mechanisms of CLE treatment.

UPLC‐TOF MS/MS analysis demonstrated that the main active compounds of CLE were catechin, quercetin, kaempferol, and their derivatives, which have been evidenced as neuroprotective phytochemicals (Costa, Garrick, Roquè, & Pellacani, 2016; Hussein, Mohamed, & Omar, 2018; Sutcliffe, Winter, Punessen, & Linseman, 2017). Moreover catechin, an essential constituents of tea polyphenol, has been evidenced in upregulation of CREB/BDNF signaling (Ding, Ma, Man, & Lv, 2017; Mi et al., 2017). Therefore, the neuroprotective effect of CNC leaves might be a combined effect of these active compounds.

In conclusion, our study demonstrated the neuroprotective effects of phytochemicals from *Camellia nitidissima* Chi leaves in H_2_O_2_‐treated SH‐SY5Y cells, and the phytochemicals of catechin, procyanidin, quercetin, kaempferol, and their derivatives might work synergistically to enhance endogenous defenses.

## 5 ETHICAL REVIEW

This study does not involve any human or animal testing.

## CONFLICT OF INTEREST

The authors declare that they do not have any conflict of interest.

## Supporting information

Supplementary Material
