# Supplementary material for: Neuroprotective effects of Camellia nitidissima Chi leaf extract in hydrogen peroxide‐treated human neuroblastoma cells and its molecule mechanisms
Source: Food Sci Nutr. 2020 Jul 22;8(9):4782–93. doi: 10.1002/fsn3.1742 (PMC7500780; doi:10.1002/fsn3.1742)
Supplement: Supplementary file 1 — Supplementary Material [file FSN3-8-4782-s001.docx]

**Materials and Methods**

1. Total Polyphenols Assay

The total phenolic content was determined by the spectrophotometric method of Folin–Ciocalteu with some modifications. The extract (1mL) was mixed with 10% Folin-Ciocalteu’s phenol reagent (2.5mL). After 3 min, the mixture was added with 1ml of 12% (w/v) Na_2_CO_3_ solution and adjusted to 25 mL with distilled water. The reaction was kept in dark for 60min at room temperature and the absorbance was measured at 765 nm. The total phenolic content was expressed as gallic acid equivalents in mg/g extract (GAEs).

2. Total Flavonoids Assay

The total flavonoids content was determined by the modified colorimetric method. The extract (0.5 mL) was mixed with 4.5 mL of 70% ethanol and subsequently with 0.3 mL of NaNO_3_ (5%) solution. After the mixture was kept for 6-min, 0.3mL of AlCl_3_ (10%) solution was added for further reaction of 6 min. Finally, 4mL NaOH (4%) was added to the mixture and the total volume was adjusted to 10 mL with 70% ethanol for measurement of absorbance at 510 nm after 15 min reaction. The total flavonoid content was expressed as rutin equivalents in mg/g extract (RTEs).

3. Antioxidant activity

The antioxidant activity of the CNC extracts was measured using DPPH and ABTS radical scavenging assays and ferric reducing antioxidant power (FRAP) assay. Briefly, for DPPH assay, 100 μL alcoholic DPPH (0.25 mM) solution and 100 μL of CNC extract or Trolox were mixed in microplate wells. The plate was oscillated for 5 sec and then incubated in dark for 30 min at room temperature. The absorbance at 517 nm was measured in a microplate reader (Molecular Devices, LLC, Sunnyvale, CA, USA). For ABTS assay, the ABTS (7.4 mM) solution was diluted with 2.6 mM potassium phosphate buffer (pH 7.4) to an absorbance of 0.700 ± 0.020 at 734 nm. 10 μL of CNC extract or Trolox and 200 μL of ABTS solution were mixed in microplate wells and kept in dark at room temperature for 6 min. The absorbance at 734 nm was measured. Trolox was used as a standard for the assays of DPPH at 2.5 to 200 𝜇m and ABTS at 25 to 2000 𝜇m, and the results were expressed as mmol Trolox equivalents per g of CNC extract samples.

For FRAP assay, the FRAP reagent was freshly prepared by mixing 300 mM of acetate buffer, 10 mM TPTZ, and 20 mM FeCl_3_ (10v : 1v : 1v). 300 μL FRAP reagent and 10 μL CNC extract or Trolox were mixed in microplate wells. The plate was oscillated for 5 sec and then incubated at 37 ℃ in dark for 30 min. The absorbance at 593 nm was measured. Aqueous solutions of Fe^2+^ at concentrations of 0.1～1 mM were used for preparation of the standard curve, and the results were also expressed as mmol Trolox equivalents per g of CNC extract samples.

**Results**

1. Phenolic contents

It has been well documented that phenolic contents including the total polyphenols and the total flavonoids were always closely correlated with antioxidant activity of botanical extracts. After the ethanolic extract was fractionated into n-hexane, ethyl acetate, and n-butanol and water soluble portions, the contents of total polyphenols and total flavonoids of different extracts ware determined.

The phenolic contents of CNC leaves extracts were listed in Table 1. The total polyphenols content ranged from 89 to 292 GAEs/g. The total polyphenols was highest in the ethyl acetate fraction and lowest in the water fraction among all extracts (292.1±9.8 and 89.9±2.8 mg GAEs/g, respectively). The total polyphenols content of ethanolic extract (229.1±3.9 mg GAEs/g) was higher than that of n-butanol fraction (186.3±1.2 mg GAEs/g) and n-hexane fraction (198.4±2.7 mg GAEs/g). Similar results were obtained for the total flavonoids content (Table 1). The total flavonoids content of CNC leaves extracts ranged from 89 to 775 mg RTEs /g. The total flavonoids of ethyl acetate fraction (774.7±26.4 mg RTEs /g) was significantly higher than that of the other extracts. These results indicate that the ethyl acetate fraction contains rich phenolic and flavonoids compounds.

**Table 1.** The contents of total polyphenols (GAEs) and total flavonoids (RTEs) in the five extracts of *C. nitidissima* Chi (CNC) leaves

| **CNC samples** | **GAEs^1^ (mg/g)** | **RTEs^2^ (mg/g)** |
| --- | --- | --- |
| Ethanolic extract | 229.1±3.9^a^ | 399.2±13.0^a^ |
| n-hexane fraction | 198.4±2.7^ab^ | 342.4±9.4^a^ |
| Ethyl acetate fraction | 292.1±9.8^c^ | 774.7±26.4^b^ |
| n-butanol fraction | 186.3±1.2^b^ | 382.6±14.1^a^ |
| Water fraction | 89.9±2.8^d^ | 88.9±4.8^c^ |

Data is expressed as mean ± standard deviation (SD) (n=3). The different letters (a, b, c, d) in same row are significantly different (*P*<0.05) according to One-way ANOVA followed by Tukey's multiple comparisons test. Results are representative of three individual experiments.

^1^ Total polyphenols contents: expressed as mg of gallic acid equivalent per g of the sample.

^2^ Total flavonoids contents: expressed as mg of rutin equivalent per g of the sample.

2. Antioxidant activity

The antioxidant activities of the CNC leaf extracts were determined by the scavenging capacities of synthetic free radicals DPPH (1, 1-diphenyl-2-picrylhydrazyl) and ABTS (2, 2’-and-bis (3-ethylbenzothiazoline-6-sulfonic acid) diammonium salt), and ferric reducing antioxidant power (FRAP). Trolox was considered as a positive control and the results were expressed as mmol Trolox equivalents per g of the sample.

As shown in Table 2, all the five extracts exhibited good antioxidant activities *in vitro.* The ethyl acetate fraction of CNC leaves (CLE) showed the best scavenging capacities of DPPH and ABTS among these five extracts, with the Trolox equivalents of 4.0±0.1 and 6.7±0.3 mmol/g, respectively. The ethanolic extract showed significantly higher scavenging capacity of DPPH and ABTS than the other three extracts. The same trend of the scavenging capacity as the phenolic contents of the five extracts was obtained. Similar in the FRAP assay, CLE showed the best ferric reducing antioxidant power, with the Trolox equivalents of 3.7±0.5 mmol/g, followed by ethanolic extract. The water fraction showed the lowest antioxidant activities in these three assays. These results indicate that the antioxidant activities *in vitro* of extracts from CNC leaves are well correlated to their phenolic contents, in consistence with previous reports.

**Table 2.** The antioxidant activities of the five extracts from *Camellia. nitidissima* Chi (CNC)

| **CNC samples** | **DPPH**  **(mmol/g)** | **ABST**  **(mmol/g)** | **FRAP (mmol/g)** |
| --- | --- | --- | --- |
| Ethanolic extract | 2.2±0.2^a^ | 3.5±0.2^a^ | 2.3±0.1^a^ |
| n-hexane fraction | 2.0±0.3^a^ | 3.0±0.1^b^ | 1.5±0.2^b^ |
| Ethyl acetate fraction | 4.0±0.1^b^ | 6.7±0.3^c^ | 3.7±0.5^c^ |
| n-butanol fraction | 2.0±0.1^a^ | 2.6±0.2^b^ | 1.8±0.1^d^ |
| Water fraction | 0.7±0.1^c^ | 1.2±0.2^d^ | 1.1±0.1^e^ |

Data are expressed as mean ± SD (n=3～6). The antioxidant activities (DPPH, ABTS and FRAP assays) are expressed as mmol Trolox equivalents per g of the sample. The different letters (a, b, c, d, e) in same row are significantly different (*P*<0.05) according to One-way ANOVA followed by Tukey's multiple comparisons test. Results are representative of three individual experiments.

3. The effects of extracts from Camellia nitidissima Chi (CNC) leaf on H_2_O_2_-induced injury in SH-SY5Y cells

In order to assess the effects of the five extracts from CNC leaves on H_2_O_2_-induced death of SH-SY5Y cells. Cells were pretreated with the extracts for 12 h, and then with H_2_O_2_ for 6 h. MTT analysis (Fig. 2) showed that pretreatment with ethyl acetate fraction (CLE, 50～150μg/ml) had the strongest effect and significantly increased the viability of H_2_O_2_-treated SH-SY5Y cells in a concentration-dependent manner. The ethanolic extract pretreatment showed significant protective effect at concentrations of 50 and 100μl/mg, but not at 150μg/ml. The other fractions showed no protective effect or even significant cytotoxicity at concentrations up to 50 μg/ml (n-hexane and ethyl acetate fractions). These results suggest that ethyl acetate fraction (CLE) may be the active fraction and the cytoprotective effects is not well co-related with the antioxidant activities *in vitro.*


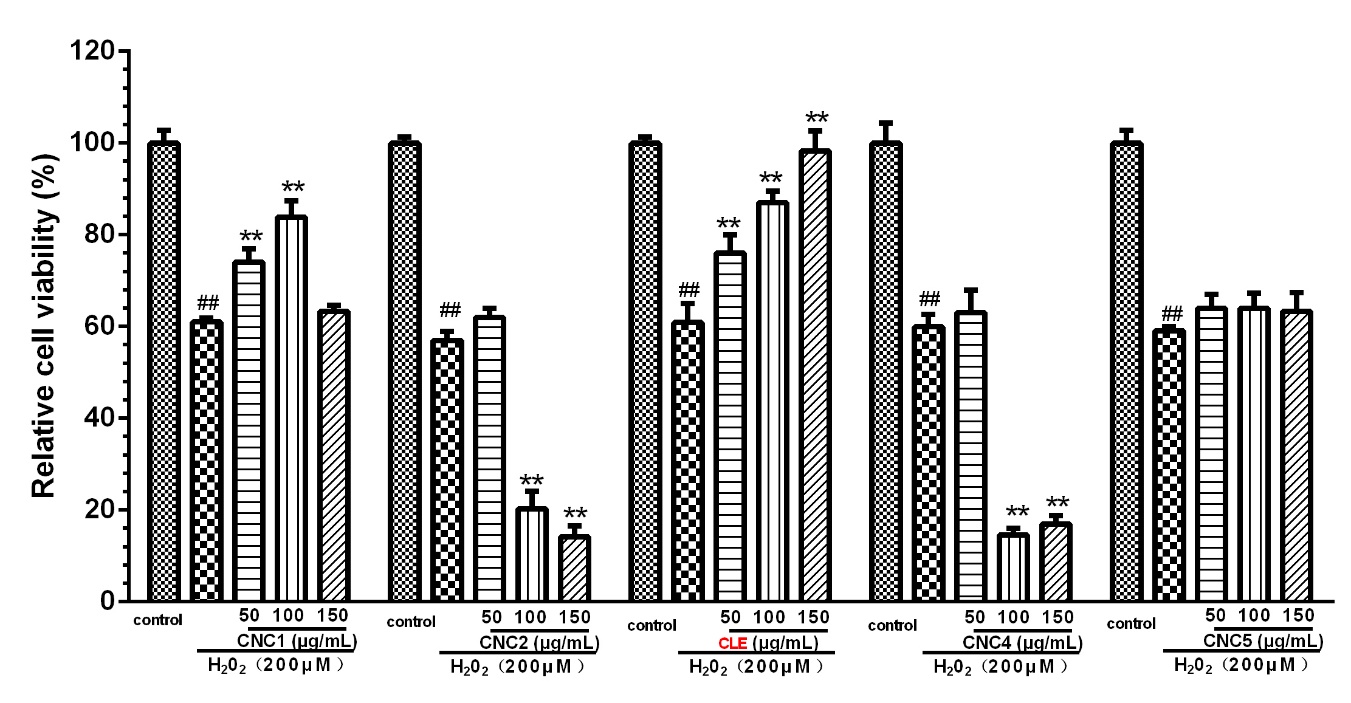


**Figure2.** Effects of five extracts from CNC leaves on viability of H_2_O_2_-treated SH-SY5Y cells by MTT assay. Each column is expressed as mean ± SD (n=6). Data are expressed as a relative percentage of the control. Samples were extracted from *C. nitidissima* Chi (CNC) leaves. CNC1: ethanolic extract; CNC2: n-hexane fraction; CLE: ethyl acetate fraction; CNC4: n-butanol fraction; CVC5: water fraction. ANOVA followed by Dunnett's test were used for statistical analysis, ^##^*P* < 0.01, compared with respective control group, ^**^*P* < 0.01, compared with respective H_2_O_2_ group. Results are representative of at least three individual experiments.
